# Peripheral Gangrene as the Initial Presentation of Systemic Lupus Erythematosus in Emergency Department

**DOI:** 10.7759/cureus.6667

**Published:** 2020-01-15

**Authors:** Zainab M Alalawi, Samar Alkenany, Fatemah Almahroos, Basmah Albloushi

**Affiliations:** 1 Emergency Medicine, King Fahd Hospital of the University, Imam Abdulrahman Bin Faisal University, Al-Khobar, SAU; 2 Emergency Medicine, College of Medicine, Imam Abdulrahman Bin Faisal University, Dammam, SAU

**Keywords:** systemic lupus erythematosus, digital gangrene, raynaud’s phenomenon, vasculitis

## Abstract

Systemic lupus erythematosus (SLE) is a chronic multisystemic autoimmune disease. Among the cutaneous manifestations of SLE, digital gangrene is considered to be very rare. This complication, which may lead to severe ischemic necrosis and amputation, is suggested to be the result of poor perfusion that is usually caused by vasculitis, vasospasm, thromboembolism, or atherosclerosis. Digital gangrene is seen mostly at a late stage of the disease proposing that a long history of SLE is a considered risk factor. Only 0.2% of patients with SLE presented initially as digital necrosis. This is a case report of a 20-year-old Saudi female who presented to the emergency room primarily with acute painful localized dry digital gangrene associated with bilateral lower limbs petechial rash. Her medical history was not suggestive of autoimmune diseases. Serology was positive for SLE. A diagnosis of SLE, lupus nephritis, and vasculitis has been established clinically and serologically. The patient adequately responded to rituximab and steroids as a medical therapy. To our knowledge, cases of acute peripheral gangrene as the initial and only presentation of SLE have rarely been documented in Emergency Medicine.

## Introduction

Systemic lupus erythematosus (SLE) is a chronic autoimmune disease of an unknown cause that can affect any organ in the body by producing autoantibodies and consequently inducing inflammation [1]. Although it can present by any means, the usual primary presentation of SLE includes arthritis, rash, and fever. Cutaneous manifestations of SLE are malar rash, alopecia, discoid lupus erythematosus, photosensitivity, livedo reticularis, and digital gangrene. Among those, digital gangrene is considered to be very rare. It occurs only in 1.3% of SLE patients [2]. There have been limited reports regarding digital dry gangrene as an initial clinical presentation of SLE. This complication, which may lead to severe ischemic necrosis and amputation, is suggested to be the result of poor perfusion that is usually caused by vasculitis, vasospasm, thromboembolism, or atherosclerosis [2].

Here, we report a rare case of a 20-year-old Saudi female, who was not known to have any medical illness and presented initially as a picture of acute peripheral digital gangrene with positive SLE serology. She was then diagnosed with SLE, lupus nephritis, and skin vasculitis. The patient adequately responded to rituximab and steroids as initial management.

## Case presentation

A 20-year-old Saudi female, who was not known to have any medical condition, presented to the emergency room of King Fahd University Hospital in Saudi Arabia, on September 20, 2019, with a one-week history of acute painful dark discoloration of the right hand. It involved the tips of the third and the fifth digits, and was associated with reddish skin lesions on bilateral lower limbs in the form of a petechial rash that was extended from the feet to the distal parts of both legs. There was no prior history of similar presentation. There was no associated history of other cutaneous manifestations such as oral ulcer, photosensitivity, malar rash, or alopecia. Before the onset of the discoloration, the patient complained of continuous subjective fever for two weeks that was partially relieved by paracetamol. Moreover, the patient gave a history of on-off Raynaud's phenomenon.

**Figure 1:**
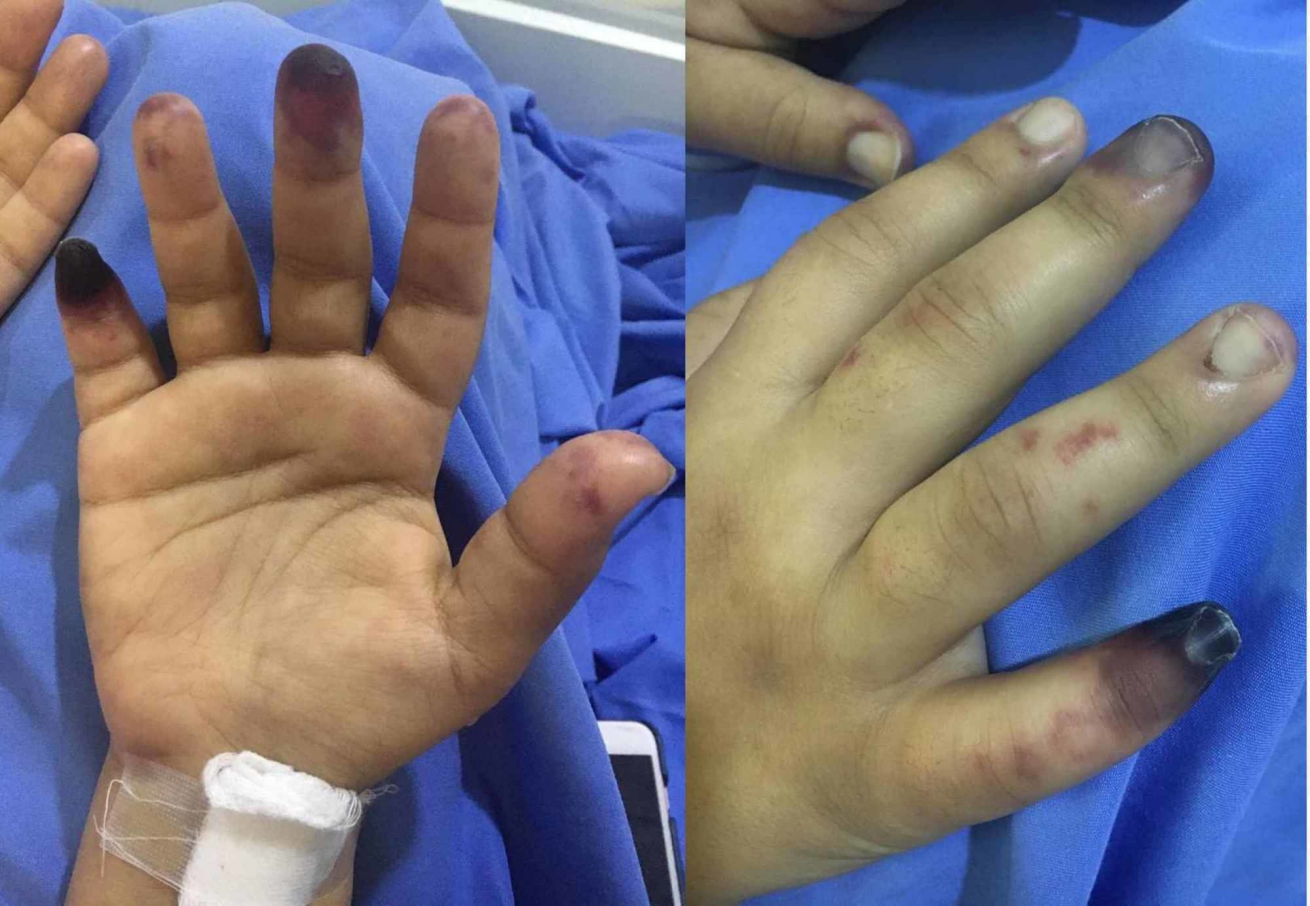
Dry gangrene located on the tips of the third and fifth digits of the right hand of a 20-year-old female.

**Figure 2:**
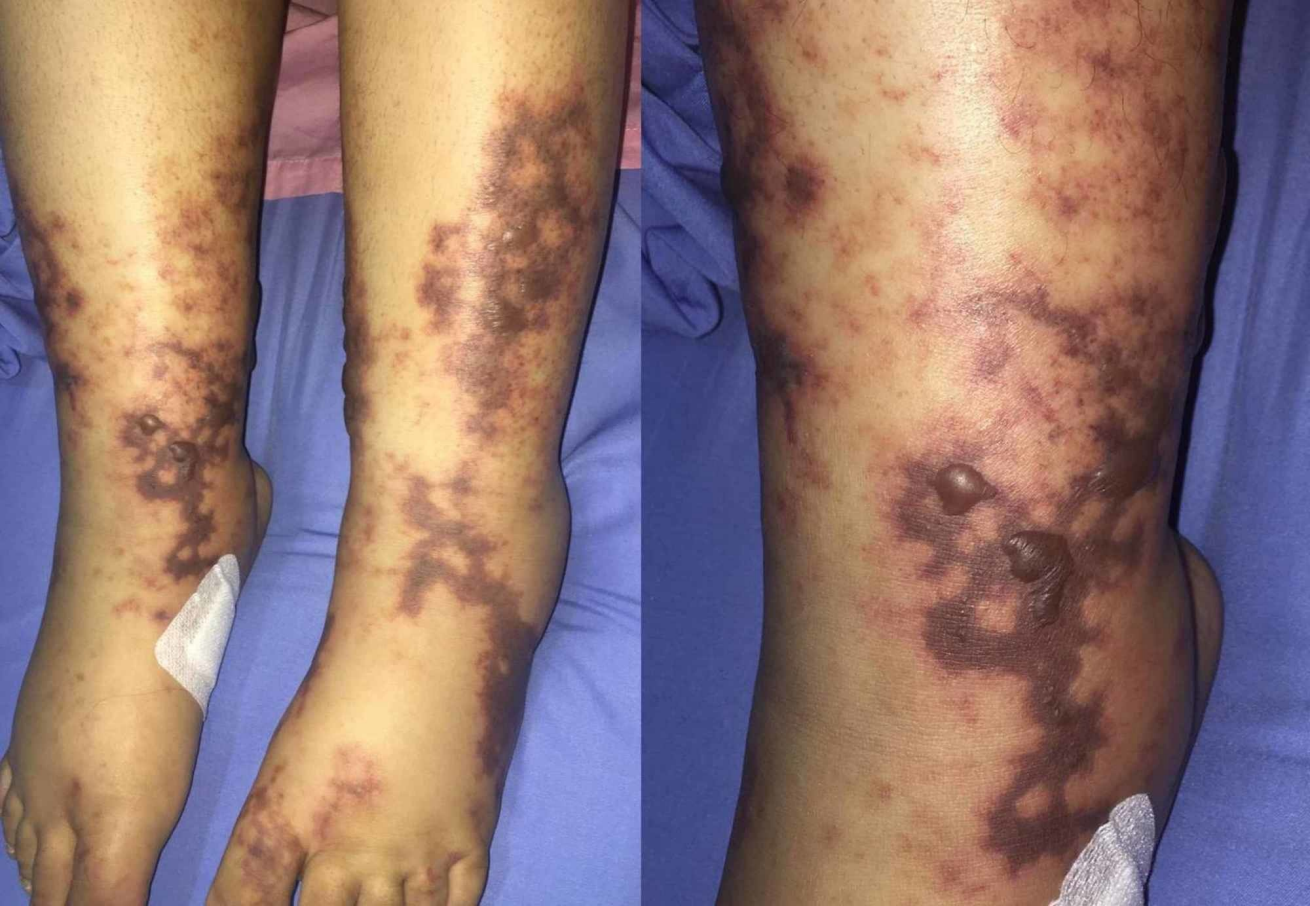
Livedo reticularis and a petechial rash over the feet and distal legs of a 20-year-old female.

On examination, the vital signs included a heart rate of 125 beats/min, a blood pressure of 129/64 mm/Hg, a respiratory rate of 20 breaths/min, and a temperature of 38°C. For her general appearance, the patient appeared mildly pale. She had palmar erythema in the right hand as well as localized dry gangrene in the tips of the third and fifth digits (Figure 1). There were purpura and livedo reticularis on the feet extending to the distal legs with hotness and tenderness (Figure 2). There was no edema or signs of deep venous thrombosis. Cardiovascular, chest, and abdomen examinations were all insignificant.

Her laboratory investigation revealed high titers of SLE-specific antibodies including anti-double-stranded DNA and anti-Smith antibody, as well as positive antinuclear antibody and low level of complement 4. The rest of the laboratory results are shown in Table 1. Doppler ultrasonography for the right lower limb veins showed that the common femoral, superficial femoral, and popliteal veins to be patent and completely compressible. However, there was evidence of subcutaneous edema over the right foot. Bilateral upper limb computed tomography angiography was done and showed patent but fairly opacified lumen of the bilateral radial and ulnar arteries. There was also an attenuated palmar arch with faint contrast opacification. The radiography of the feet was reported as normal. 

**Table 1:** Laboratory results of 20-year-old female presented with acute digital gangrene.

Laboratory Result					
Test	Result	Normal level	Test	Result	Normal level
White blood cells, x10^9^/l	4.9	4.0-10.5	Myeloperoxidase antibodies	Negative	Negative
Hemoglobin, g/dl	7.7	12.6-17.7	Anti-Smith antibodies	Positive	Negative
Platelet count, x10^9^/l	299	140-415	Anti-beta-2 glycoprotein antibodies IgM	Negative	Negative
Erythrocyte sedimentation rate, mm/hr	19	0-20	Anti-beta-2 glycoprotein antibodies IgG	Positive	Negative
C-reactive protein, mg/dl	2.8	<3	Anti-Sjogren's antibodies SSA	Positive	Negative
Lactic acid, mmol/l	1.5	<2	Anti-Sjogren's antibodies SSB	Negative	Negative
Malaria smear	Negative	Negative	Cardiolipin IgM	Negative	Negative
Coombs test	Positive	Negative	Cardiolipin IgG	Negative	Negative
C4 complement, mg/dl	2	20-40	Anti-Jo-1	Negative	Negative
Anti-double-stranded DNA	Positive	Negative	Anti-ribonucleoprotein RNP	Positive	Negative
Antinuclear antibody	Positive	Negative	Anti-cyclic citrullinated peptidase antibody	Positive	Negative
Protease 3 antibodies	Negative	Negative	Rheumatoid factor	Negative	Negative

## Discussion

Peripheral digital gangrene has been described as a rare manifestation of SLE. It occurs only in 1.3% of SLE patients [2]. In 2009, a total of 2,684 patients with lupus were screened, and only 18 patients were found to have digital necrosis [1]. On a large study done on a cohort of lupus patients, seven out of 485 patients had evidence of digital gangrene [3]. Out of 344 patients, 20 cases of lupus digital gangrene had also been reported among the Indian population in 2009 [4].

Furthermore, peripheral digital gangrene as the first presenting symptom of SLE is rarely reported. Only 0.2% of patients with SLE present initially as digital gangrene [5]. Former reports declared that it is seen mostly at a late stage of the disease. In their study that was conducted in 1962, Dubois and Arterberry found that the latent period between diagnosing SLE and developing digital gangrene was from 1 to 18 years [2]. Rosato et al. claimed that acute digital gangrene is never the first presentation of SLE [6]. Our patient’s presentation is not only rare but also goes against the usual picture of SLE for she did not show any prior features of SLE.

Our patient's age was consistent with the age that was mostly reported in the literature of peripheral digital gangrene. If it were to appear, SLE-related digital gangrene is more commonly seen during adulthood. It is rare to have digital gangrene in children below the age of five years. Yet, it is worth mentioning that there has been one report of digital gangrene in infancy [7].

Former literature suggested that for SLE patients, a medical history of Raynaud’s phenomenon is a predictive factor for developing digital necrosis [1]. However, Dubois and Arteberry reported one of five cases of SLE-related gangrene where Raynaud’s phenomenon preceded the digital necrosis [2]. Among seven patients with SLE coexisting with digital gangrene, Alarcon-Segovia and Osmundson described three patients who were found to have Raynaud's phenomenon prior to peripheral gangrene [8].

Other predictive factors that were described in the literature include a long-standing history of SLE and elevated C-serum reactive protein (CRP) [9]. A cohort study that was done on 2,684 patients showed that CRP level was distinctively high in patients who had digital gangrene [1]. Yet, looking to our patient, her CRP was within normal range (Table 1) and she did not have a prior history of SLE. The only known risk factor she had for developing digital gangrene was a history of Raynaud’s phenomenon.

One form of SLE that was described frequently in patients with diganital gangrene is antiphospholipid syndrome (APS). Of all patients with APS, digital gangrene was found in 3.3%-7.5% of the cases [10]. APS is characterized by the presence of antiphospholipid antibodies, which contribute to thrombus formation and consequently gangrene development [1]. Antiphospholipid antibodies are anticardiolipin antibody, anti-beta-2-glycoprotein I antibodies (IgG or IgM), and lupus anticoagulant assay [11]. The clinical features of APS are livedo reticularis, chronic ulcers, arterial or venous thrombosis, recurrent abortions, and neurological symptoms [10]. Although she had a positive anti-beta-2 glycoprotein antibody IgG (Table 1), our patient was not diagnosed with APS for she did not meet the clinical criteria of APS diagnosis described by UpToDate (Wolters Kluwer Health, Waltham, MA) and did not have a confirmed thrombus [12]. Moreover, beta-2-glycoprotein antibodies IgG should present on two or more occasions, at least 12 weeks apart [12].

The suggested treatment of digital gangrene in the literature includes corticosteroids, immunosuppressants, lipid-lowering agents, and anticoagulation [1]. The former reports supported the use of cyclophosphamide as an immunosuppressive agent. However, judging by the minimizing of the dry digital gangrene, our patient showed clinical improvement after a single dose of 1,000 mg IV of rituximab.

## Conclusions

We highlight this rare presentation of severe vasculitis exacerbation that led to digital gangrene in a patient who had never been diagnosed with SLE. We are pleased to report that our patient responded well to the initial treatment, and she showed clinical improvement of her symptoms. Her condition is being adequately followed up.
